# P-1289. Trends, Treatments, and Outcomes in Vibrio Species Infections: The UTMB Galveston Experience

**DOI:** 10.1093/ofid/ofae631.1470

**Published:** 2025-01-29

**Authors:** Grant Bradley Torres, Kimberley C Brondeel, Andi J Cummins, Camila Franco Mesa, Joshua Klinnert, Ludwik Branski, Joseph Patrik Hornak, Miguel M Cabada, David Reynoso

**Affiliations:** University of Texas Medical Branch, Galveston, Texas; The University of Texas Medical Branch, Galveston, Texas; University of Texas medical branch, Galveston, Texas; University of Texas Medical Branch, Galveston, Texas; University of Texas Medical Branch, Galveston, Texas; The University of Texas Medical Branch, Galveston, Texas; University of Texas Medical Branch, Galveston, Texas; University of Texas Medical Branch, Galveston, Texas; The University of Texas Medical Branch, Galveston, Texas

## Abstract

**Background:**

*V. vulnificus*, *V. parahaemolyticus*, and other species thrive in coastal water of areas such as the East Coast of Texas. There are few large-scale, single-institution retrospective studies investigating Vibrio spp. infections. Our study aims to determine the clinical presentation, management, and outcomes of infections caused by Vibrio spp. treated at the University of Texas Medical Branch (UTMB) in Galveston.

**Methods:**

A retrospective chart review was conducted for patients with culture-positive Vibrio spp. samples at the UTMB from 2016-2023. Primary outcomes include clinical characteristics, antibiotic treatment, surgical management, and outcomes of Vibrio spp. infections. Secondary outcomes include demographic data, comorbidities, and causative agents for Vibrio spp. infections. This study was performed in collaboration with the infectious disease department and plastic surgery division at UTMB.

**Results:**

25 patients treated for Vibrio-related infections were included. Patient demographic data is as follows: 24 (96%) were Caucasian, 23 (92%) were non-Hispanic, 21 (84%) were male, and the average age was 62.2 years. Nearly all cases occurred in Galveston County and the surrounding area, with 18 (72%) associated with water/aquatic exposure. 13 (52%) patients noted contact with marine life shortly before admission, comprising most of the 12 (48%) patients noting underlying injury before symptom onset. 8 patients had localized soft tissue infections, while 17 developed systemic infections. Most systemic manifestations developed secondary to soft tissue infection. Surgical intervention was required in nearly half of the patients. *V. vulnificus* was noted in 18 patients (mostly systemic), and *V. parahaemolyticus* was noted in 7 patients. The most common empiric antibiotics were Vancomycin, Piperacillin-Tazobactam, and Doxycycline—followed by Ceftriaxone, and Clindamycin.

**Conclusion:**

In areas along the coast with warm seawater or brackish water, infections due to Virbio spp. warrant hefty consideration and prompt treatment—as over 2/3 of our patients quickly developed systemic infection and nearly half required surgical intervention. This is especially true for patients with open wounds exposed to these aquatic environments, and those in contact with marine life.

**Disclosures:**

**Joseph Patrik Hornak, MD**, Innoviva Specialty Therapeutics: Advisor/Consultant|Innoviva Specialty Therapeutics: Advisor/Consultant

